# Modified trapeziectomy with ligament reconstruction tendon interposition for the treatment of advanced thumb carpometacarpal arthritis: A case report

**DOI:** 10.1097/MD.0000000000009665

**Published:** 2018-02-02

**Authors:** Ji Peng You, Lu Lu, Cong Jie Li, Bao Ren, Tao Wang

**Affiliations:** a Department of Orthopaedics; b Department of Emergency; c Department of Anesthesiology; d Department integrated Chinese and Western medicines. Affiliated Hospital of Hebei University, Baoding, China.

**Keywords:** flexor carpi radialis, modified, thumb carpometacarpal arthritis, trapeziectomy

## Abstract

**Rationale:**

Thumb carpometacarpal (CMC) arthritis is a common disease. Various procedures have been described for the treatment of advanced thumb CMC arthritis. This essay shows a CMC arthritis case treated by modified trapeziectomy with ligament reconstruction tendon interposition (LRTI).

**Patient concerns:**

A 53-year-old Chinese female complained of pain and swelling at the base of the left thumb for 10 years. Visual analog scale (VAS) for thumb was 7 points, Disabilities of Arm, Shoulder and Hand (DASH) score was 51 points, and Kapandji score was 6 points before surgery. Preoperative range of motion (ROM) for radial abduction and volar abduction were 63°and 62°, respectively. Grip power was 15.3 kg and key-pinch power was 1.8 kg before operation. Preoperative waist flexion power was 20.9 kg. Hand x-ray showed left thumb CMC arthritis in Eaton stage III and the height of the trapezial space was 10 mm.

**Diagnoses:**

She was diagnosed with left thumb CMC arthritis (Eaton III stage).

**Interventions:**

The patient underwent modified trapeziectomy with LRTI. After exposing and removing trapezium, and a hole from the dorsal base to the center of the articular surface was drilled. Then we cut the whole flexor carpi radialis and divided it into 2 halves. Afterward, we passed one-half through the hole and tied it to the other part and sutured them. The rest tendon was then tied continuously and sutured. Then we rolled it up into the space where previous trapezium was located.

**Outcomes:**

Two years after operation, pain and swelling relieved and no recurrence of the clinical symptoms occurred. VAS, DASH, and Kapandji score were 2, 22, 7 points, respectively. ROM for radial abduction and volar abduction were 79° and 78°, respectively. Furthermore, grip power was 22.7 kg and key-pinch power was 3.8 kg. Waist flexion power was 20.0 kg. Hand x-ray showed that the height of the trapezial space was 9.8 mm.

**Lessons:**

Modified trapeziectomy with LRTI in treatment of advanced thumb CMC arthritis had a satisfactory efficacy. This new procedure not only prevents thumb sinking, but also provides enough support for thumb.

## 1 Introduction

Thumb carpometacarpal (CMC) arthritis is the second most common degenerative joint disease of the hand, accounting for 11% and 33% of men and women as previous studies reported,^[1,2]^ respectively. This difference of incidence in gender may be related to level of hormonal.^[3]^ Thumb CMC arthritis could lead to pain, laxity, and weakness of the CMC joint.^[4]^ As we know, the thumb CMC joint is a biconcave–convex saddle joint and its articulation includes the first metacarpal of the thumb and the trapezium carpal bone, which enables CMC joint motion in 3 different planes: adduction–abduction, flexion–extension, and axial rotation.^[5–7]^

According to the Eaton stage, conservative therapy was effective for patients with Eaton stage I, however, as for patients in Eaton stage II–IV, surgical procedure, including trapezial excision, ligament reconstruction with or without tendon interposition, was used as the best choice.^[3–5]^ Badia^[8]^ performed a retrospective study on patients with stage II receiving osteotomy and found that osteotomy was only useful in patients who have not experienced complete articular cartilage loss. Avisar et al^[9]^ conducted a long-term follow-up on trapeziectomy with abductor pollicis longus (APL) arthroplasty for thumb CMC arthritis. The results showed that trapeziectomy with APL arthroplasty had a satisfactory efficacy for patients with Eaton stage II–IV in a long follow-up.

Increasing articles reported on trapeziectomy with ligament reconstruction tendon interposition (LRTI) treating thumb CMC arthritis. Here we show modified trapeziectomy with LRTI in treatment for advanced thumb CMC arthritis.

## 2 Consent

The present study was approved by ethics committee of the Affiliated Hospital of Hebei University. There is no need to obtain informed consent from the patient because all the data were collected and analyzed anonymously.

## 3 Case report

**Figure 1:**
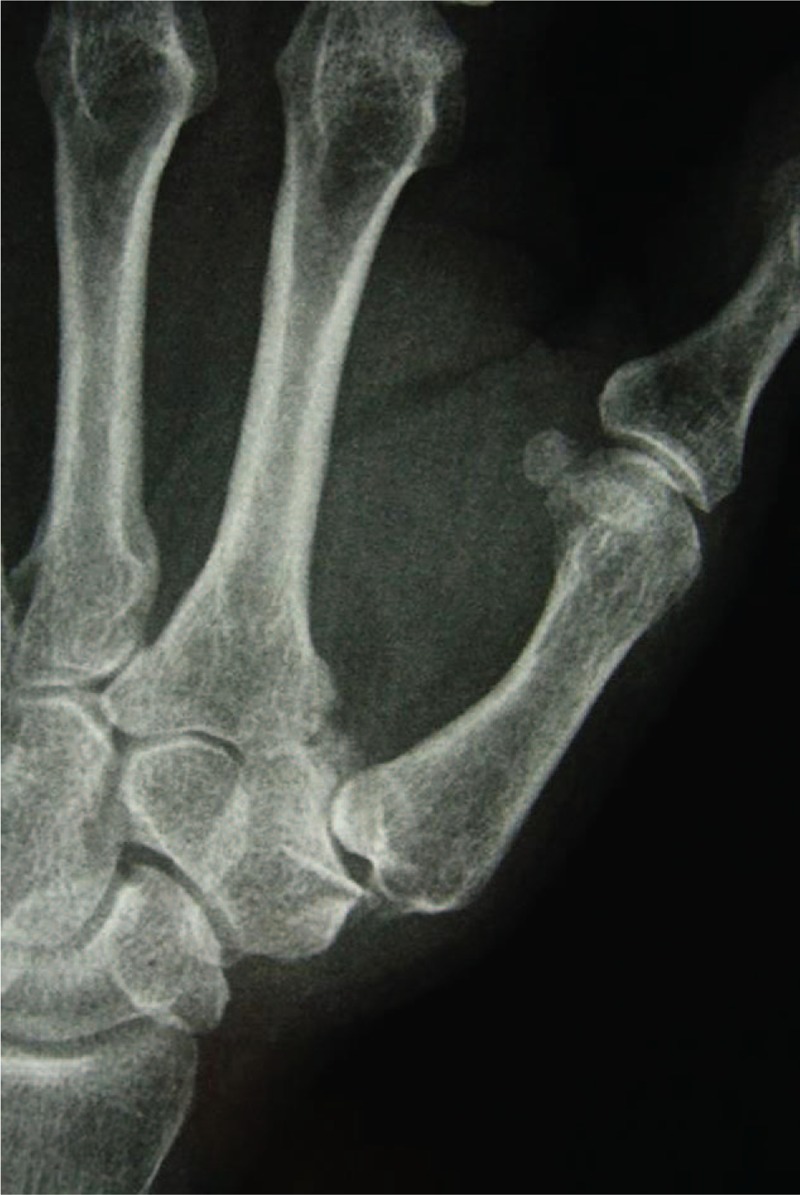
Hand x-ray showing the height of the trapezial space to be10 mm and left thumb CMC arthritis. CMC = carpometacarpal.

**Figure 2:**
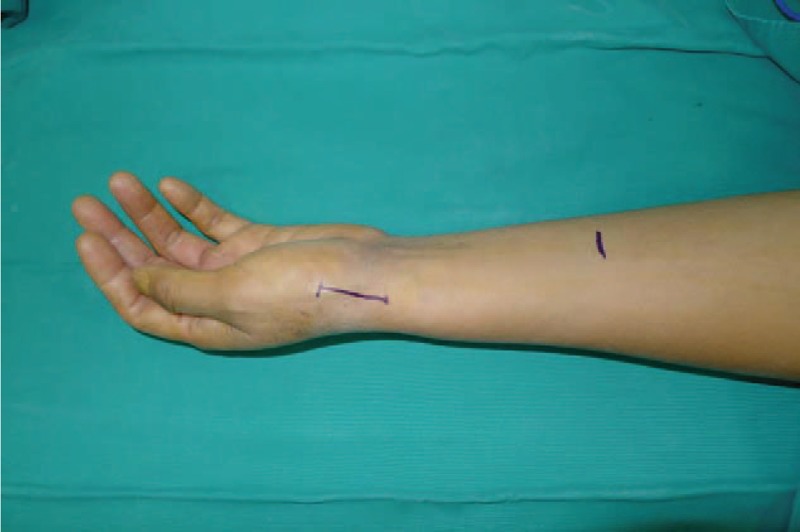
An incision is made at the dorsal thumb metacarpal bone.

**Figure 3:**
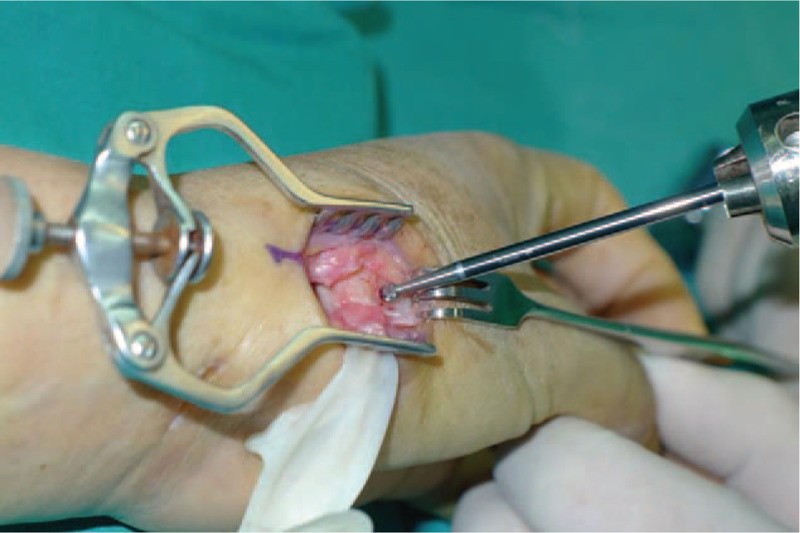
Exposed trapezium and excised piecemeal.

**Figure 4:**
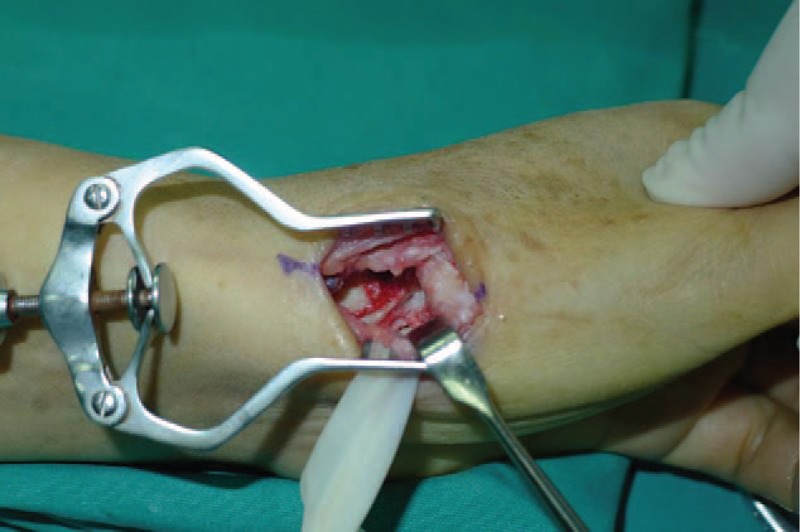
Removed trapezium.

**Figure 5:**
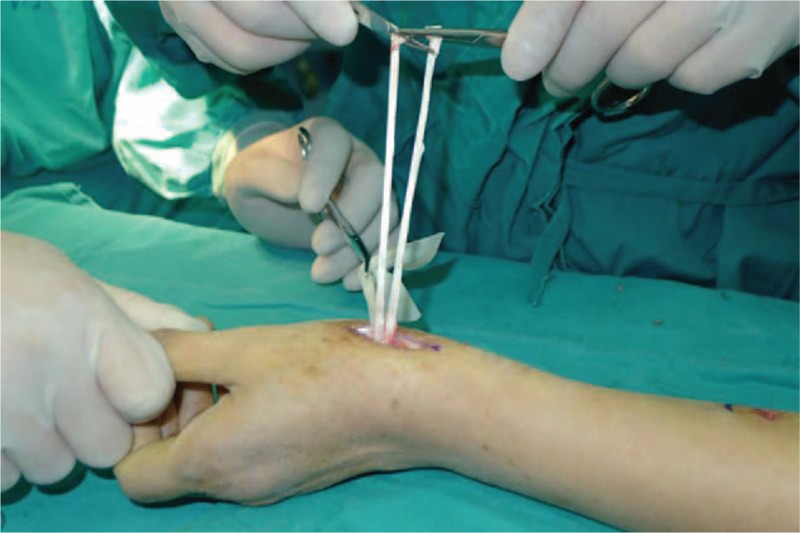
Cut flexor carpi radialis in half.

**Figure 6:**
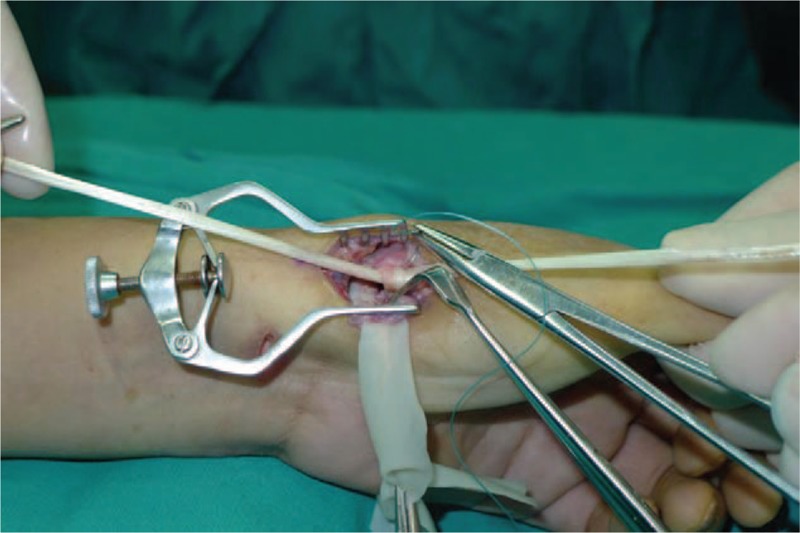
We passed one-half of FCR through the hole and tied with the other and sutured. FCR = flexor carpi radialis.

**Figure 7:**
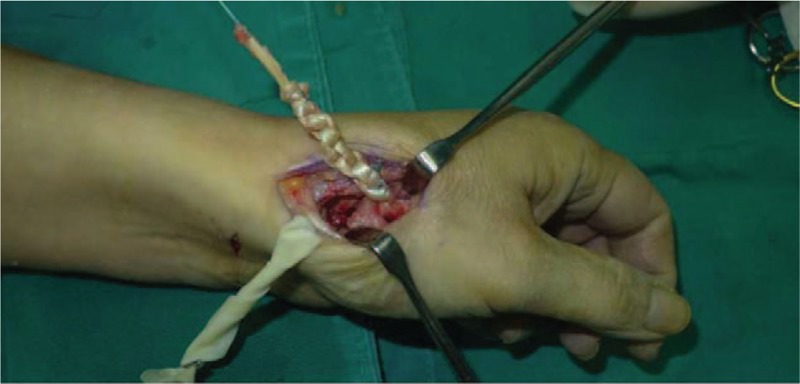
We sewed continuously and rolled FCR up. FCR = flexor carpi radialis.

**Figure 8:**
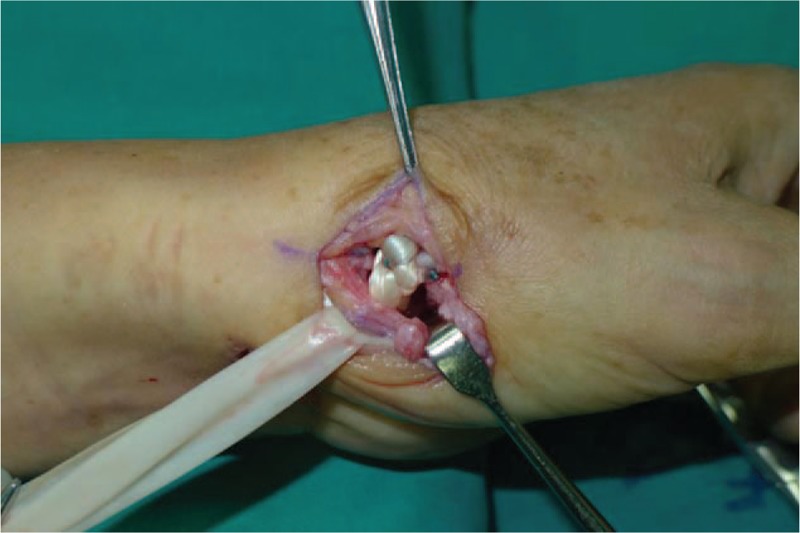
We put flexor carpi radialis into room where trapezium is located in.

**Figure 9:**
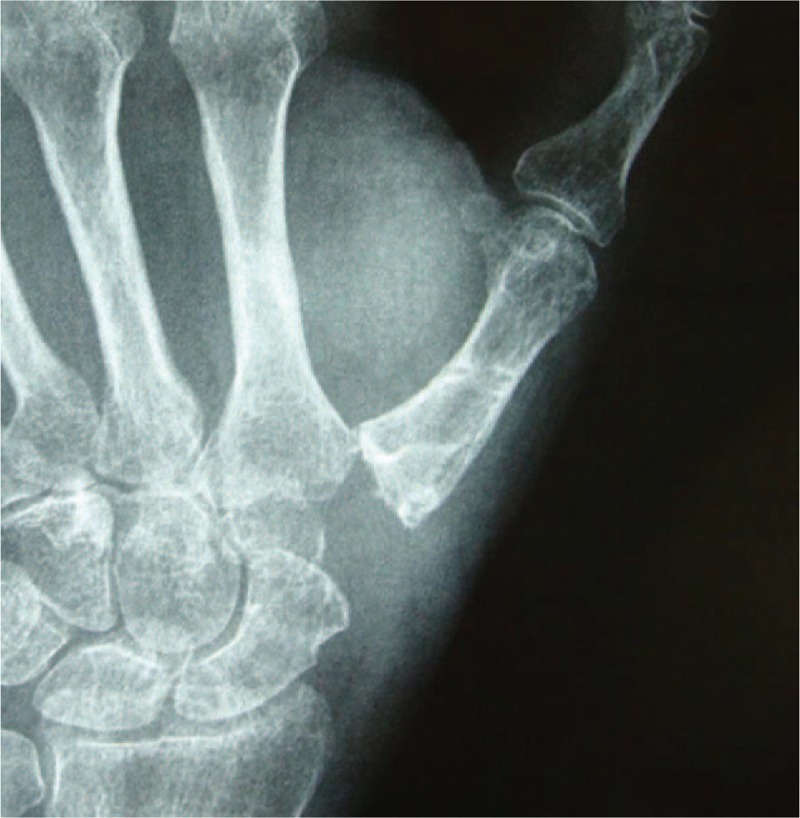
Hand x-ray showing the height of the trapezial space to be 9.8 mm.

A 53-year-old adult woman presented with a complaint of pain and swelling at the base of the left thumb for 10 years. Preoperative visual analog scale (VAS), Disabilities of Arm, Should and Hand (DASH) score, and Kapandji score for thumb were 7, 51, and 6 points, respectively. Preoperative range of motion (ROM) for radial abduction and volar abduction were 63° and 62°, respectively. Additionally, preoperative grip power was 15.3 kg and key-pinch power was 2.6 kg. Preoperative waist flexion power was 20.9 kg. Hand x-ray showed the height of the trapezial space to be 10 mm and left thumb CMC arthritis in Eaton stage III, as shown in Figure 1. The patient was diagnosed with left thumb CMC arthritis with Eaton stage III. We performed modified trapeziectomy with LRTI on this patient. We made a longitudinal incision at the dorsal thumb metacarpal bone shown in Figure 2 The capsule of the CMC joint was longitudinally incised, while paying attention to protecting superficial branch of radial nerve, and the trapezium was exposed and excised piecemeal. After removing trapezium, we could see flexor carpi radialis (FCR) (Figs. 3 and 4) and then drilled a hole from the dorsal base to the center of the articular surface. We made a tiny incision in the dorsal forearm and identified FCR. We cut whole FCR off from the transitive place between tendon and muscle and picked it out from space created by trapeziectomy. Like in Figure 5, we longitudinally split FCR into 2 halves and passed one-half of it through the hole and tied it to the other part and sutured them. Then the rest tendon was tied continuously, as shown in Figure 6. We sewed every knot and rolled up like a ball (Fig. 7), then put it into room where previous trapezium was located (Fig. 8). We sutured the tendon ball to the volar capsule and ligament and closed the capsule to prevent it from escaping. We conducted gypsum fixation for a week and external fixation for 2 weeks. From fourth week, this patient did functional exercise. According to a 2-year follow-up, the patients showed decreased VAS score from 7 to 1 point. Score showed improvement from 6 to 7 points for Kapandji and from 51 to 22 points for DASH. Radial abduction markedly improved from 63° to 79° and volar abduction from 62° to 78°. Power obviously improved from 15.3 to 22.8 kg for grip power and from 1.9 to 3.8 kg for key-pinch power. Waist flexion power was 20.0 kg. Hand x-ray showed the height of the trapezial space to be 9.8 mm (Fig. 9).

## 4 Discussion

Due to complex anatomy, the thumb CMC joint is a commonly affected site for arthritis in hands. However, it remains controversial to treat thumb CMC arthritis with Eaton stage II–IV. The management for thumb CMC joint varies from simple trapeziectomy to numerous ligament reconstruction surgeries. Of ligament reconstruction surgeries, FCR is commonly used to reinforce first metacarpal bone. Saehle et al^[10]^ compared APL with FCR as a ligament reconstruction to treat thumb CMC arthritis and concluded that the latter one had better outcomes in the fields of key-pinch and grip strengths. Previous studies reported using radial half of the FCR to insert in the base of the second metacarpal.^[1,3]^ But some authors deemed that using half of the FCR was unable to supply adequate tension during reconstruction, which could cause the tendon to slip and pain.^[2]^

Here we showed a case that a 53-year-old adult woman complained of pain and swelling at the base of the left thumb for 10 years. We adopted a new surgical procedure, modified trapeziectomy with LRTI, for that patient. We cut total FCR off and divided it into halves and put one-half through the hole from the dorsal base to the center of the articular surface at the base of the metacarpal bone to tie with the other, as shown in Figures 5 to 7. Then we rolled it up and put it into room where previous trapezium was located (Fig. 8). According to a 2-year follow-up, VAS (from 7 to 1 points) and DASH (from 51 to 22 points) scores dramatically reduced. Scores improved from 6 to 7 points for Kapandji. Radial abduction markedly improved from 63° to 79° and volar abduction from 62° to 78°. Power obviously improved from 15.3 to 22.7 kg for grip power, and from 1.9 to 3.8 kg for key-pinch power. Additionally, waist flexion power slightly weakened from 20.9 to 20.0 kg. From the above, the data proved that modified trapeziectomy with LRTI was an effective treatment for thumb CMC arthritis while cutting whole FCR did not markedly affect the wrist flexion power. Hand x-ray showed that the trapezial just sank from 10 mm preoperatively to 9.8 mm at a 2-year follow-up (Fig. 9). There were following merits. First, as we have known the insertion of FCR is at the base of the second metacarpal, we indirectly hold the thumb and index metacarpal together which is more likely to be able to stabilize first metacarpal. Second, the tendon-to-bone fashion is able to stabilize first metacarpal and is also beneficial to recover key-pinch power. Third, the method that one-half tied continuously and sutured with the other, which allows enough tendon ball to provide adequate support for thumb metacarpal, can delay sinking which causes short deformity and diminished strength. Although this procedure provides several advantages, it has some limitations. First, it needs a longer follow-up to prove its efficacy; second, we cut whole FCR off, but the wrist flexion strength is not significantly declined; third, we need more cases to assess this procedure.

In conclusion, thumb CMC arthritis is a common disease in clinic. Increasing articles had been reporting various surgeries to treat thumb CMC arthritis. Modified trapeziectomy with LRTI is an effective treatment for advanced thumb CMC arthritis. It not only stabilizes first metacarpal, but also provides enough support for it, which makes its sinking process slower. We provide a new method for surgeons when facing thumb CMC arthritis and we need further study to observe efficacy in longer term follow-up.
