# Modified trapeziectomy with ligament reconstruction tendon interposition for the treatment of advanced thumb carpometacarpal arthritis: A case report: Erratum

**DOI:** 10.1097/MD.0000000000010040

**Published:** 2018-02-23

In the article, “Modified trapeziectomy with ligament reconstruction tendon interposition for the treatment of advanced thumb carpometacarpal arthritis: A case report”,^[1]^ which appeared in Volume 97, Issue 5 of *Medicine*, the corresponding author information should appear as:

Bao Ren, Department of Orthopaedics, Affiliated Hospital of Hebei University, Baoding, China (e-mail: docwangspine@163.com).
